# Acute sleep deprivation has no lasting effects on the human antibody titer response following a novel influenza A H1N1 virus vaccination

**DOI:** 10.1186/1471-2172-13-1

**Published:** 2012-01-04

**Authors:** Christian Benedict, Maria Brytting, Agneta Markström, Jan-Erik Broman, Helgi Birgir Schiöth

**Affiliations:** 1 Department of Neuroscience, Uppsala University, Uppsala, Sweden; 2 Department of Virology, Swedish Institute for Infectious Disease Control, Solna, Sweden; 3 Department of Neuroscience, Psychiatry, Sleep Medicine Center, University Hospital, Uppsala, Sweden

## Abstract

**Background:**

Experimental studies in humans have yielded evidence that adaptive immune function, including the production of antigen-specific antibodies, is distinctly impaired when sleep is deprived at the time of first antigen exposure. Here we examined the effects of a regular 24- hour sleep-wake cycle (including 8 hours of nocturnal sleep) and a 24-hour period of continuous wakefulness on the 7-week antibody production in 11 males and 13 females in response to the H1N1 (swine flu) virus vaccination. The specific antibody titer in serum was assayed by the hemagglutination inhibition test on the days 5, 10, 17, and 52 following vaccination.

**Results:**

In comparison to the sleep group, sleep-deprived males but not females had reduced serum concentration of H1N1-specific antibodies five days after vaccination, whereas antibody titers at later time points did not differ between the conditions.

**Conclusions:**

These findings concur with the notion that sleep is a supportive influence in the very early stage of an adaptive immune response to a viral antigen. However, our results do not support the view that acute sleep deprivation has lasting effects on the human antibody titer response to influenza vaccination.

## Background

The lack of time to sleep is a hallmark of modern living, and it is commonly assumed that in the long run this makes us unwell. This assumption is supported by experimental data showing that acute sleep deprivation decreases or decelerates the production of antigen- specific antibodies if sleep is interrupted in the night following the vaccination [1,2], indicating that poor sleep patterns potentially counteract the process of effective adaptive immune responses. Sleep is assumed to regulate immune function primarily by fostering adaptive immune responses [3-5]. In the present study, we investigated a 7-week antibody titer in males and females in response to a novel influenza A H1N1 virus (swine flu) vaccination and measured the effects of sleep in those who had a single night of sleep deprivation versus no sleep deprivation.

## Results

### Hemagglutination inhibition antibody titer against the H1N1 virus

**Figure 1:**
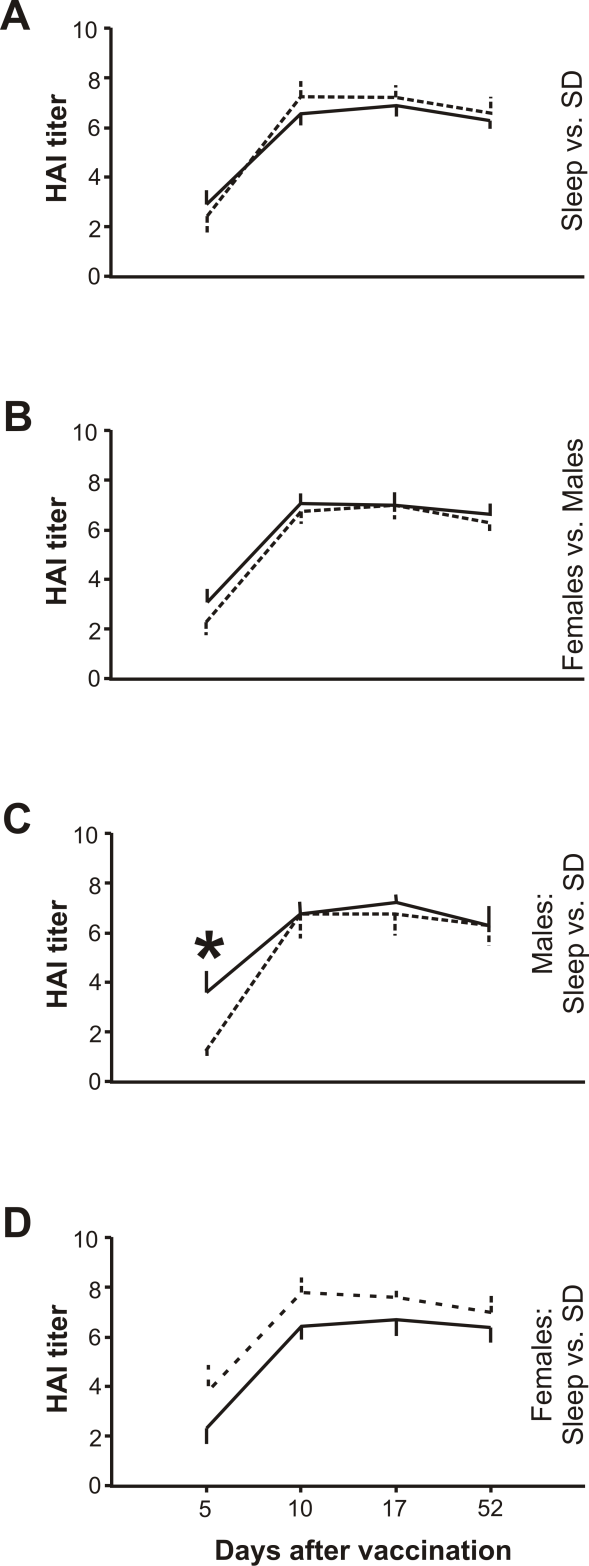
**The effects of sleep compared with those of sleep deprivation (SD) on the antibody titer in males and females in response to the novel influenza A H1N1 virus vaccination**. The serum antibody response following the vaccination against H1N1 was assayed by the hemagglutination inhibition test (HAI) as previously described (4). The higher the HAI value, the higher the detectable serum antibody titer specific for H1N1 virus. **A**, antibody response split by sleep (solid line, N = 13) vs. sleep deprivation (dashed line, N = 11). **B**, antibody response split by females (solid line, N = 13) vs. males (dashed line, N = 11). **C**, antibody response in males split by sleep (solid line, N = 5) vs. sleep deprivation (dashed line, N = 6). **D**, antibody response in females split by sleep (solid line, N = 8) vs. sleep deprivation (dashed line, N = 5). Differences between (sub)groups were analyzed using the Kruskal-Wallis test, with Mann-Whitney U post hoc testing, and a P value ≤ 0.05 was considered statistically significant. All data are presented as means ± SEM.

Overall, the antibody production did not differ between treatments and/or sexes. (P ≥ 0.302 for all Kruskal-Wallis comparisons, Figure 1AB). However, five days after vaccination, the antibody response associated with sleep deprivation was approximately 60% lower in males than that measured in those of the sleep group (P ≤ 0.050, two-tailed Mann-Whitney test; P ≤ 0.037 for the Kruskal-Wallis comparison; Figure 1C). In contrast, the immune response in women was generally not influenced by sleep deprivation (P ≥ 0.171 for all Kruskal-Wallis comparisons, Figure 1D).

### Sleep

The EEG recordings revealed a normal sleep pattern (in min ± SEM; Total sleep time: 470 ± 27; Slow-wave sleep, 145 ± 14; Rapid-eye movement sleep, 81 ± 11). Data gathered using sleep diaries (recording the time of sleep) during the first 10 days after H1N1 vaccination ensured the continuation of regular sleep start times (ie., between 22:00 PM and 8:00 AM the next morning) in both conditions.

## Discussion

Acute sleep deprivation impaired the early immune response to H1N1 virus in males, despite prolonged periods of sleep recovery after antigen exposure. In contrast, in females, the production of antibodies specific for the swine flu virus was not influenced by sleep loss. These results underline the relevance of sleep for supporting immune functions [3-5].

Our results suggest that acute sleep deprivation is associated with a delayed induction of adaptive immune response to H1N1 virus in males that is consistent with previous data. That is, interrupted sleep at the time of first virus exposure is followed by a slower increase in specific antibody titers relative to that following undisturbed sleep [1]. Factors important for an effective adaptive immune response show very concordant changes under sleep deprivation [4,6]. For instance, the production of growth hormone normally peaks during early sleep periods [7], and this effect is suppressed by sleep deprivation [8]. This hormone stimulates immune function directly in that many immune cells possess receptors for growth hormone [9]. Further, the secretion of cytokines involved in the adaptive immune response is downregulated by sleep loss (e.g. Interleukin-2 and Interleukin-7; [10,11]). Such hormonal changes associated with interrupted sleep might have contributed to the decelerated immune response to H1N1 that was observed in males but not females in our study. Interestingly, in a recently published study, a lipopolysaccharide-induced immune response following acute sleep loss has been shown to induce distinct sex differences inasmuch as sleep-deprived females showed increased production of proinflammatory cytokines compared to response in sleep-deprived males [12]. However, there are also other candidate mechanisms that may account for the impaired antibody titer response in sleep-deprived males seen on day 5. For instance, compared with sleep, sleep loss is associated with a striking decrease in the number of myeloid dendritic cell precursors [13]. These cells play a major role for the initiation of adaptive immune responses [14]. Further it might be that the impaired effect of sleep loss on the early antibody titer response to the H1N1 vaccination was due to a delayed migration of antibody titer producing B cells into the blood.

At the first glance, the clinical relevance of our findings is somewhat questionable because the only difference in specific antibody titers is 5 days after vaccination in males only and titers even out thereafter, which in the end results in same possible protection in sleep deprived and regular sleep subjects. In contrast, other studies with a similar study design showed profound effects of sleep deprivation on specific antibody titers at later time points, e.g. a 2-fold higher hepatitis A titer at day 28 post immunization in regular sleep subjects as compared to sleep deprived men [2]. Nevertheless, in a sort of external validation of our results, previous observations revealed that partial sleep deprivation in the time of vaccination against a seasonal influenza virus induced differences in specific antibody titers during early periods (ie., after 10 days) but not during late periods (ie., after 28 days) of the adaptive immune response [1]. One explanation for the discrepancy in results among these studies could be that the effect size of sleep deprivation on adaptive immune responses is antigen- specific.

## Limitations

Due to the similarity of the antigen with other influenza strains there might be the possibility that the history of influenza vaccination plays a profound role in the response to H1N1 vaccine. In this respect, the major shortcoming of the present study is that no HAI titers were determined before vaccination to exclude possible cross-reactivity of antibodies possibly already present. Although it was vital that participants reported, in a thorough interview, to have been free of major influenza-like symptoms in the past 6 months, we thus cannot exclude that there were any baseline differences in HAI titers before vaccination. However, given the age of the subjects (around 20 years) it is unlikely that they had previously been exposed to a similar H1N1 until the recent pandemic. Sleep deprivation did not influence the antibody titer response to H1N1 in females. Moreover, there were no differences between the sleep and sleep deprivation conditions in terms of circulating concentrations of H1N1 antibodies at time points later than day 5. These negative findings should be interpreted with caution inasmuch as they could be caused by the low detection power associated with the relatively low sample size of our study. Thus, these results indisputably require further validation in more studies. Finally, although we obtained EEG measures in five subjects of the sleep group, and thus quantitatively assured that subjects did sleep, we cannot rule out that those whose sleep were not recorded may have deviated from their habitual sleep patterns. However, based on the sleep diaries, all participants, including those who did not undergo EEG recordings, did not report subjective disturbances in their sleep habits.

## Conclusions

Our results suggest that sleep deprivation in the night after vaccination may influence very early phases of an adaptive immune response, as indicated by the lower antibody titer response in sleep-deprived males measured 5 days after vaccination against the H1N1 virus. However, since antibody titers at later time points did not differ between the sleep and sleep deprivation conditions, our results do not support the view that acute sleep deprivation has lasting effects on the human antibody titer response to influenza vaccination.

## Methods

### Subjects

24 healthy non-smoking students participated in the experiment (born after 1986; body-mass- index, range 20-25 kg/m^2^, Table 1). They were assigned to two groups: 'sleep' (n = 13; 5 males) or 'sleep deprivation' (n = 11; 6 males). The groups did not differ in terms of age or body weight. Acute illness was excluded by physical examination and routine laboratory investigation. During an interview before the study, all participants reported having a regular sleep-wake rhythm (i.e. participants' main sleep periods were between 10:00 PM and 8:00 AM the next morning; no shift work) and none were taking medication (including analgesics such as acetaminophen). Further, to be included in the study it was vital that participants reported, in a thorough interview, to have been free of major influenza-like symptoms in the past 6 months (e.g., high fever, chest congestion, vomiting). The study was approved by the Regional Ethical Review Board (EPN) in Uppsala, Sweden. All participants gave written informed consent and were paid for enrolling in the study.

**Table 1:** Subjective data

		Sleep group	Sleep deprivation group
**Number of subjects**	All	13	11
	Women	8	5
	Men	5	6
**Age (in years)**	All	20.6 ± 0.4	20.4 ± 0.5
	Women	21.5 ± 0.4	20.2 ± 0.7
	Men	19.2 ± 0.4	20.5 ± 0.7
**Body weight (in kg)**	All	65.4 ± 4.9	64.6 ± 5.7
	Women	56.9 ± 6.0	50.2 ± 7.6
**C-reactive Protein (mg/L)**	Men	79.0 ± 3.7	76.7 ± 4.2
	All	0.98 ± 0.26	1.22 ± 0.32
	Women	0.97 ± 0.25	1.21 ± 0.38
**Ig G (g/L)**	Men	0.99 ± 0.60	1.24 ± 0.53
	All	10.61 ± 0.63	10.5 ± 0.41
	Women	10.61 ± 0.85	10.56 ± 0.70
**Ig A (g/L)**	Men	10.60 ± 1.04	10.38 ± 0.53
	All	1.99 ± 0.25	2.10 ± 0.41
	Women	1.82 ± 0.23	2.13 ± 0.39
	Men	2.28 ± 0.23	2.07 ± 0.55
**Ig M (g/L)**	All	1.30 ± 0.16	1.22 ± 0.18
	Women	1.46 ± 0.23	1.54 ± 0.29
	Men	1.05 ± 0.16	0.96 ± 0.18

All variables are presented with mean ± S.E.M. Three days before vaccination, circulating levels of C-reactive protein and immunglobulin subclasses (A, G, M) were analyzed by normal laboratory routine. No statistical differences were found between the groups (assessed by Mann-Whitney-U-tests).

### Experimental design and procedure

The experimental time schedule is presented in Table 2. All subjects were vaccinated against swine flu (0.5-mL intramuscular injection, Pandemrix, GlaxoSmithKline, UK) at 7:30 AM on the morning of 27th November 2009. They were not informed about their respective group assignment until 8:00 PM the same day. The electroencephalography (EEG) of five students of the sleep group was continuously recorded in the night after vaccination using an ambulatory amplifier (Embla, Flaga hf, Iceland), and polysomnography (including EMG and EOG) was performed according to standard criteria [15]. Sleep stages were determined off-line by an experienced scorer blinded to the study hypothesis. The subjects in the wake group remained in the sleep laboratory and did not sleep until 6:00 PM the following day. To keep subjects awake, they were provided with a selection of movies, games, and books and were continuously observed by investigators. Further, the lights were on (~800 lux) and the subjects were provided with regular meals. On the day of vaccination and the day after, students of both conditions were not allowed to consume caffeine-containing beverages.

**Table 2:** Experimental time schedule.

Experimental day	Date	Measurements
Day -3	2009-11-24	Health assessment
Day 0	2009-11-27	H1N1 vaccination
	2009-11-28	Sleep diary
	2009-11-29	Sleep diary
	2009-11-30	Sleep diary
	2009-12-01	Sleep diary
Day 5	2009-12-02	Sleep diary & Serum antibody titer
	2009-12-03	Sleep diary
	2009-12-04	Sleep diary
	2009-12-05	Sleep diary
	2009-12-06	Sleep diary
Day 10	2009-12-07	Sleep diary & Serum antibody titer
Day 17	2009-12-14	Serum antibody titer
Day 52	2010-01-18	Serum antibody titer

Blood was sampled at ~8:00 AM in the morning on days 5, 10, 17, and 52 after the vaccination and stored at -80°C until assay. Hemagglutination inhibition (HAI) antibody titers against the H1N1 virus were determined in duplicate. Serum samples were treated with receptor-destroying enzyme and then incubated at 56°C for 30 min. HAI assays were performed at a starting dilution of 1:10, with subsequent serial -fold dilutions (maximum dilution level, 1:5120). A seroprotective titer for HAI was defined as 1:40. Chicken erythrocytes (0.5%) were used for the agglutination. All samples from a single participant were tested simultaneously and run in duplicate.

### Statistical analysis

For statistical reasons, the dilution levels deriving from hemagglutination inhibition (HAI) assay were transformed into degrees of dilution. For instance, a HAI dilution level of 1:10 corresponds a degree of dilution of 1 (maximum dilution level, 1:5120 = degree of dilution of 10). Differences between groups were analyzed using Kruskal-Wallis nonparametric analysis with Mann-Whitney U post hoc testing, and a P value ≤ 0.05 was considered statistically significant. Treatment effects were also evaluated separately for the male and female subgroups. All data are presented as means ± SEM.

## Authors' contributions

CB, AM, and JB carried out the experiments. MB carried out the immunoassays. All authors contributed to writing. CB, AM, JB, and HS designed the study. All authors read and approved the final manuscript.
